# High prevalence of m.1555A > G in patients with hearing loss in the Baikal Lake region of Russia as a result of founder effect

**DOI:** 10.1038/s41598-024-66254-z

**Published:** 2024-07-03

**Authors:** Tuyara V. Borisova, Aleksandra M. Cherdonova, Vera G. Pshennikova, Fedor M. Teryutin, Igor V. Morozov, Alexander A. Bondar, Olga A. Baturina, Marsel R. Kabilov, Georgii P. Romanov, Aisen V. Solovyev, Sardana A. Fedorova, Nikolay A. Barashkov

**Affiliations:** 1 Laboratory of Molecular Biology, Institute of Natural Sciences, M.K. Ammosov North-Eastern Federal University, Kulakovskogo 46, 677013 Yakutsk, Russia; 2 Laboratory of Molecular Genetics, Yakut Science Centre of Complex Medical Problems, Yaroslavskogo 6/3, 677000 Yakutsk, Russia; 3 SB RAS Genomics Core Facility, Institute of Chemical Biology and Fundamental Medicine, Siberian Branch of the Russian Academy of Sciences, Prospekt Akademika Lavrentieva 8, 630090 Novosibirsk, Russia; 4 Novosibirsk State University, 630090 Novosibirsk, Russia

**Keywords:** Hearing loss, *MT-RNR1*, m.1555A > G, A5b, Eastern Siberia, Russia, Medical genetics, Inner ear

## Abstract

Mitochondrial forms account approximately 1–2% of all nonsyndromic cases of hearing loss (HL). One of the most common causative variants of mtDNA is the m.1555A > G variant of the *MT-RNR1* gene (OMIM 561000). Currently the detection of the m.1555A > G variant of the *MT-RNR1* gene is not included in all research protocols. In this study this variant was screened among 165 patients with HL from the Republic of Buryatia, located in the Baikal Lake region of Russia. In our study, the total contribution of the m.1555A > G variant to the etiology of HL was 12.7% (21/165), while the update global prevalence of this variant is 1.8% (863/47,328). The m.1555A > G variant was notably more prevalent in Buryat (20.2%) than in Russian patients (1.3%). Mitogenome analysis in 14 unrelated Buryat families carrying the m.1555A > G variant revealed a predominant lineage: in 13 families, a cluster affiliated with sub-haplogroup A5b (92.9%) was identified, while one family had the D5a2a1 lineage (7.1%). In a Russian family with the m.1555A > G variant the lineage affiliated with sub-haplogroup F1a1d was found. Considering that more than 90% of Buryat families with the m.1555A > G variant belong to the single maternal lineage cluster we conclude that high prevalence of this variant in patients with HL in the Baikal Lake region can be attributed to a founder effect.

## Introduction

Hearing loss (HL) is one of the most common congenital diseases. The prevalence of congenital and childhood HL in the world is estimated at 1.33 per 1000 newborns^1^. It is known that up to 50% of cases of congenital HL have a hereditary cause^1,2^. Currently more than 120 genes associated with HL are known^1–3^. About 70% of hereditary causes of HL are nonsyndromic and 30% are syndromic^4^. At the same time, approximately 75% of all cases of nonsyndromic HL occur in autosomal-recessive, 10–15% in autosomal-dominant and 1–2% in X-linked recessive and mitochondrial forms^1,2^. Mitochondrial forms of HL are associated with a pathogenic variants in the genes: *MT-RNR1* (OMIM:561000), *MT-TS1* (OMIM:590080), *MT-CO1* (OMIM:516030), *MT-TH* (OMIM:590040), *MT-ND1* (OMIM:51600), *MT-TL1* (OMIM:590050), *MT-TE* (OMIM:590025) and *MT-TK* (OMIM:590060), which can lead to both isolated HL and various syndromes, when HL can be combined with the endocrine (diabetes mellitus and deafness, OMIM:520000) and nervous system pathologies (MERRF syndrome, OMIM:545000 and MELAS syndrome, OMIM:540000)^5^. Despite the low proportion of mitochondrial forms of HL, their role in the etiology of HL was described before the autosomal-recessive form of HL caused of pathogenic variants in the *GJB2* gene (DFNB1A, OMIM: 220290). In 1993 Fischel-Ghodsian and colleagues reported discovery of the m.1555A > G variant in the *MT-RNR1* gene encoding mitochondrial 12S rRNA in patients with aminoglycoside antibiotics induced HL (deafness, aminoglycoside-induced, OMIM: 561000.0001)^6–8^.

Currently, there are several hypotheses regarding the pathogenicity mechanism of the m.1555A > G variant of the *MT-RNR1* gene. One of the hypotheses suggests that this variant can exhibit a pathogenic effect without modulating factors^9–11^. Since the adenine (A) to guanine (G) substitution in 1555 position of the *MT-RNR1* gene leads to a change in the conservative A-site (aminoacyl-tRNA acceptor site) of 12S rRNA, this can lead to reading errors during the synthesis of oxidative phosphorylation proteins^11^. Another hypothesis is related to the modulating effect of aminoglycoside antibiotics. This assumption is based on the specific ability of the aminoglycosides to bind to the A-site of the 16S bacterial ribosome and thus selectively disrupt the synthesis of prokaryotic proteins without affecting eukaryotic ribosomes due to structural differences^12,13^. The A > G substitution at position 1555 of human 12S rRNA leads to a new C–G base pairing, which leads to similarity with the A-site of bacterial 16S rRNA, which is a target for aminoglycoside antibiotics^14^. Recently, using the methods of cryo-electron microscopy with chemical crosslinking/mass spectrometry, and in silico modeling, it was shown that the A > G substitution at position 1555 changes the secondary structure of human mitochondrial 12S rRNA, but does not affect folding, which indicates a greater role of the modulating factors in the pathogenicity of the m.1555A > G variant^15,16^.

Previously, to determine the most likely genetic forms of HL in the Republic of Buryatia, located in the Baikal Lake region of Russia (Eastern Siberia), a segregation analysis was carried out in 17 Buryat and 18 Russian families with hereditary history of HL^17^. This analysis suggested that the presumably hereditary cases of HL in Russian families (SF = 0.25 ± 0.07, at t = 0.64) have been segregating by the autosomal-recessive type of inheritance^17^. However, in Buryat families, the obtained segregation frequency of the HL (SF = 0.35 ± 0.05, at t = 0.38) was higher than theoretically expected for autosomal-recessive (SF_0_ = 0.25) and was lower than for autosomal-dominant type of inheritance (SF_0_ = 0.50). This result indicated the presence of other forms of HL in Buryat families that did not segregate with autosomal types of inheritance^17^. Subsequent molecular-genetic testing for the most common autosomal-recessive form of HL (DFNB1A, OMIM: 220290) confirmed that the contribution of causative variants of the *GJB2* gene to the etiology of HL in Buryat patients was only 5.1% (one of the lowest rates in the world), while in Russian patients the contribution was 28.9%^18^, which was comparable with the results of segregation analysis^17^.

In this regard, the aim of this study is to analyze of the mitochondrial variant m.1555A > G in the *MT-RNR1* gene in patients with HL in the Republic of Buryatia.

## Results

### Contribution of the m.1555A > G variant of the *MT-RNR1* gene to the etiology of the HL in 165 patients in Republic of Buryatia

**Figure 1:**
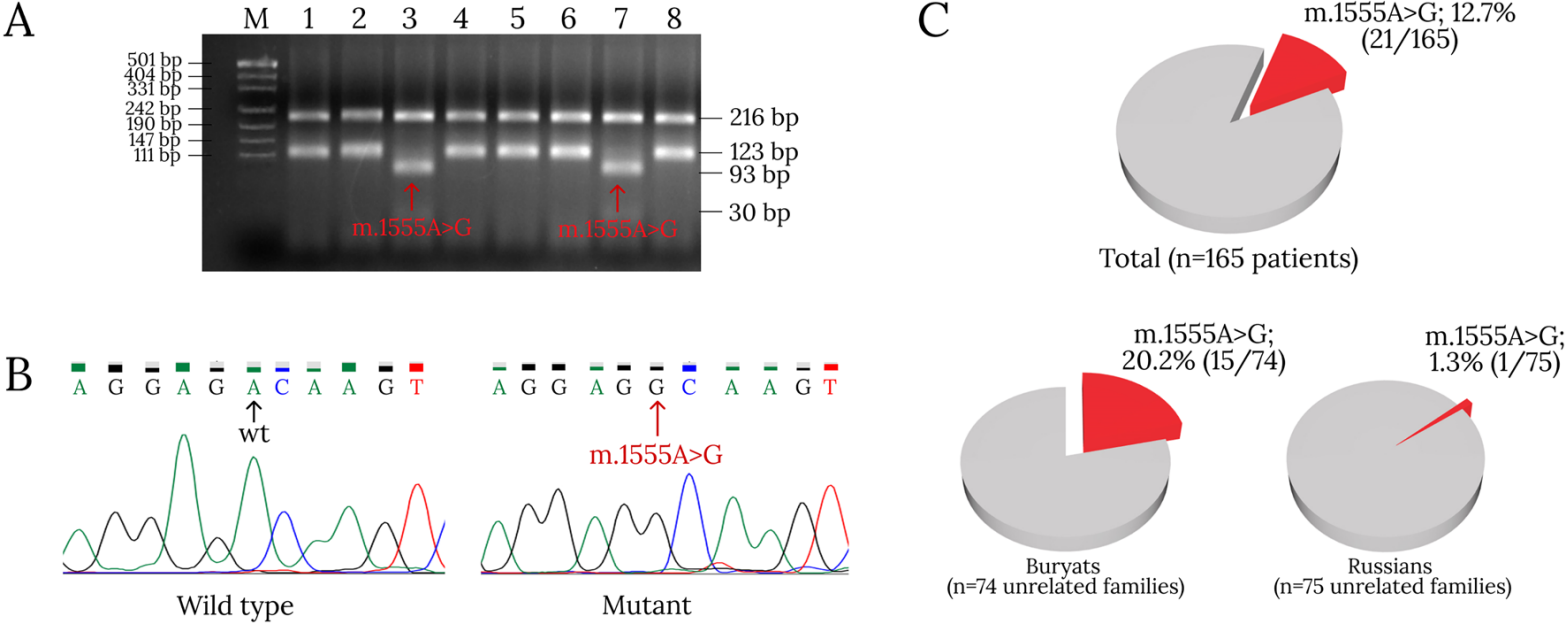
Detection of the m.1555A > G variant of the *MT-RNR1* gene and its contribution to the etiology of HL in the Republic of Buryatia. (**A**)—Detection of the m.1555A > G variant of the *MT-RNR1* gene in 3% agarose gel by PCR–RFLP analysis with use of *Hae*III: M—marker PUc19/Msp*I*, lanes 1, 2, 4–6, 8—normal (wt), lanes 3 and 7—m.1555A > G (original electrophoregram presented in supplementary Fig. S1); (**B**)—Sanger sequencing of the *MT-RNR1* gene fragment; (**C**)—The contribution of the m.1555A > G variant of the *MT-RNR1* gene in patients with HL is calculated for all patients (21 out of 165 patients), the proportion of the m.1555A > G variant of the *MT-RNR1* gene, depending on ethnicity is calculated for unrelated families.

The m.1555A > G variant of the *MT-RNR1* gene in the homoplasmic state was detected in 21 out of 165 studied patients with HL (Fig. 1). All 165 patients with HL were previously tested for the presence of pathogenic variants in the *GJB2* gene associated with autosomal-recessive deafness, type 1A (DFNB1A, OMIM 220290)^18^. No causative variants in the *GJB2* gene, including del(GJB6-D13S1830), del(GJB6-D13S1854) and del(GJB2-D13S175) were found in patients with the m.1555A > G variant in the *MT-RNR1* gene. The average age of onset of HL in patients with the m.1555A > G variant of the *MT-RNR1* gene was 2.7 years. In 85.8% of patients (n = 18) with this variant, bilateral profound sensorineural HL was confirmed. In the other 14.2% patients (n = 3), the severe degree of the HL was detected. In 23.8% of patients (n = 5) with the m.1555A > G variant we found a history of aminoglycoside use (Table S1). In this study, the total contribution of the m.1555A > G variant of the *MT-RNR1* gene to the etiology of HL in the Republic of Buryatia was 12.7%. However, there were significant differences between two main ethnic groups of patients: a higher proportion of this pathogenic variant was found in Buryat patients (20.2%) than in Russian patients (1.3%) (Fig. 1).

### Genetic-epidemiological analysis of the identified mitochondrial form of HL in the Republic of Buryatia

The prevalence of mitochondrial HL caused by the m.1555A > G variant of the *MT-RNR1* gene in the Republic of Buryatia was 0.2 per 10,000 (Table 1). The highest prevalence was found in the southern regions with the maximum accumulation in the Dzhidinskii district, where its prevalence was 4.5 per 10,000. The lowest prevalence (from 0.02 to 0.75 per 10,000) of this form of deafness was registered in the city of Ulan-Ude and four northern districts of the Republic of Buryatia (Mukhorshibirsky, Kizhinginsky, Khorinsky and Kurumkansky districts) (Table 1).

**Table 1:** The prevalence of mitochondrial HL caused by the m.1555A > G variant of the *MT-RNR1* gene in the Republic of Buryatia.

#	Administrative units	Population	Number of patients with the m.1555A > G	Prevalence per 10,000
1	City of Ulan-Ude	437,565	1	0.0229
2	City of Severobaikalsk	24,233	–	-
3	Barguzinsky municipal district	20,250	–	-
4	Bauntovsky Evenki municipal district	8,252	–	-
5	Bichursky municipal district	21,504	2	0.9301
6	**Dzhidinsky municipal district**	**22**,**021**	**10**	**4.5411**
7	Yeravninsky municipal district	17,027	–	-
8	Zaigraevsky municipal district	50,726	–	-
9	Zakamensky municipal district	24,556	–	-
10	Ivolginsky municipal district	64,862	–	-
11	Kabansky municipal district	51,780	–	-
12	Kizhinginsky municipal district	14,798	1	0.6758
13	Kurumkan municipal district	13,254	1	0.7545
14	Kyakhtinsky municipal district	32,238	3	0.9306
15	Muisky municipal district	8,970	–	-
16	Mukhorshibirsky municipal district	22,044	1	0.4536
17	Okinsky municipal district	5,323	–	-
18	Pribaikalsky municipal district	24,217	–	-
19	Severo-Baikalsky Municipal District	10,717	–	-
20	Selenginsky municipal district	41,433	–	-
21	Tarbagatai municipal district	25,600	–	-
22	Tunkinsky municipal district	20,645	–	-
23	Khorinsky municipal district	16,573	1	0.6034
The Republic of Buryatia		978,588	20	0.2044
City of Chita*		334,427	1	0.0299

The district with the highest prevalence is highlighted in bold; *— patient with m.1555A > G variant, who was born in Chita, is not included in the genetic-epidemiological analysis.

### Analysis of mtDNA haplogroups in patients with the m.1555A > G variant of the *MT-RNR1* gene

**Figure 2:**
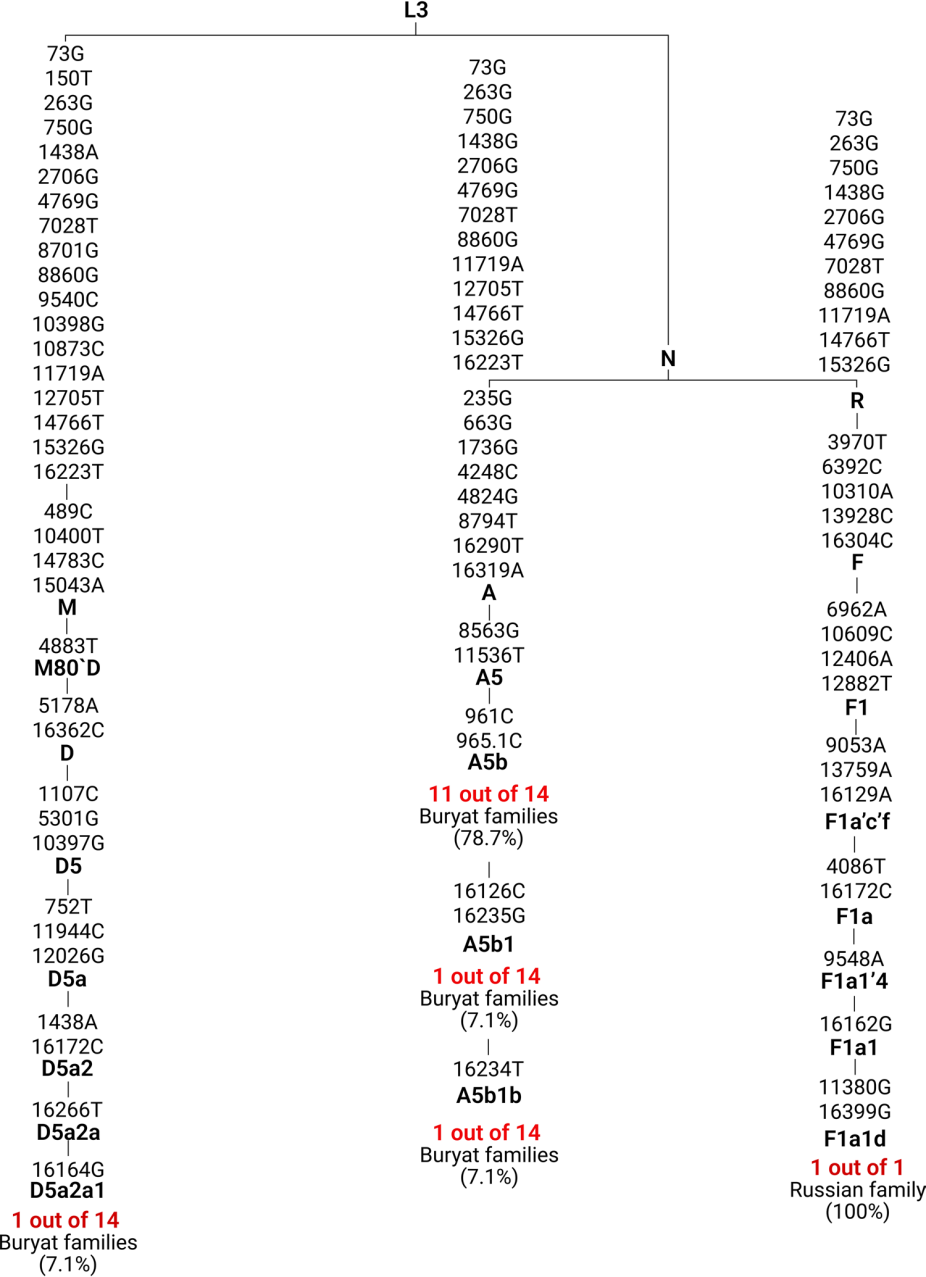
Topology of mtDNA haplogroups in 15 families with the m.1555A > G variant of the *MT-RNR1* gene.

Analysis of mitochondrial DNA haplogroups in 15 unrelated families with the m.1555A > G variant of the *MT-RNR1* gene showed that A5b sub-haplogroup was present in 13 families (86.7%), the D5a2a1 sub-haplogroup in one family (6.7%), and sub-haplogroup F1a1d also in one family (6.7%) (Fig. 2). Interestingly, that among Buryat families with the m.1555A > G variant we found a whole cluster of the A5b sub-haplogroup lineages consisting of several subclades (A5b, A5b1 and A5b1b) with frequency of 92.9%, the minor lineage (D5a2a1) was detected with frequency 7.1%. In one Russian family with the m.1555A > G variant F1a1d sub-haplogroup was found (Fig. 2).

## Discussion

In the present study, we carried out molecular-genetic screening for the m.1555A > G variant of the *MT-RNR1* gene in mitochondrial DNA in 165 patients with HL from the Republic of Buryatia, located in the Baikal Lake region of Russia. The m.1555A > G variant of the *MT-RNR1* gene contributed to the etiology of HL in 12.7% of the examined patients (21 out of 165). Notably, a high proportion of Buryat patients (20.2%) had the m.1555A > G variant compared to Russian patients (1.3%). It should be noted that this variant was not identified in the neighboring regions of South Siberia among Tuvinian (0/220) and Altaian (0/93) patients with HL^19,20^. However, the m.1555A > G variant has been identified in the northern parts of the Eastern Siberia, among Yakut (1/108) and Even (4/23) patients with HL^21–23^. This variant also has been found in Russian patients with HL from European part of Russia (4/102 and 1/122)^21,24^. In general, the prevalence of this variant in Russia is estimated at 1.18% (11/928) (Table S2)^20–24^.

**Figure 3:**
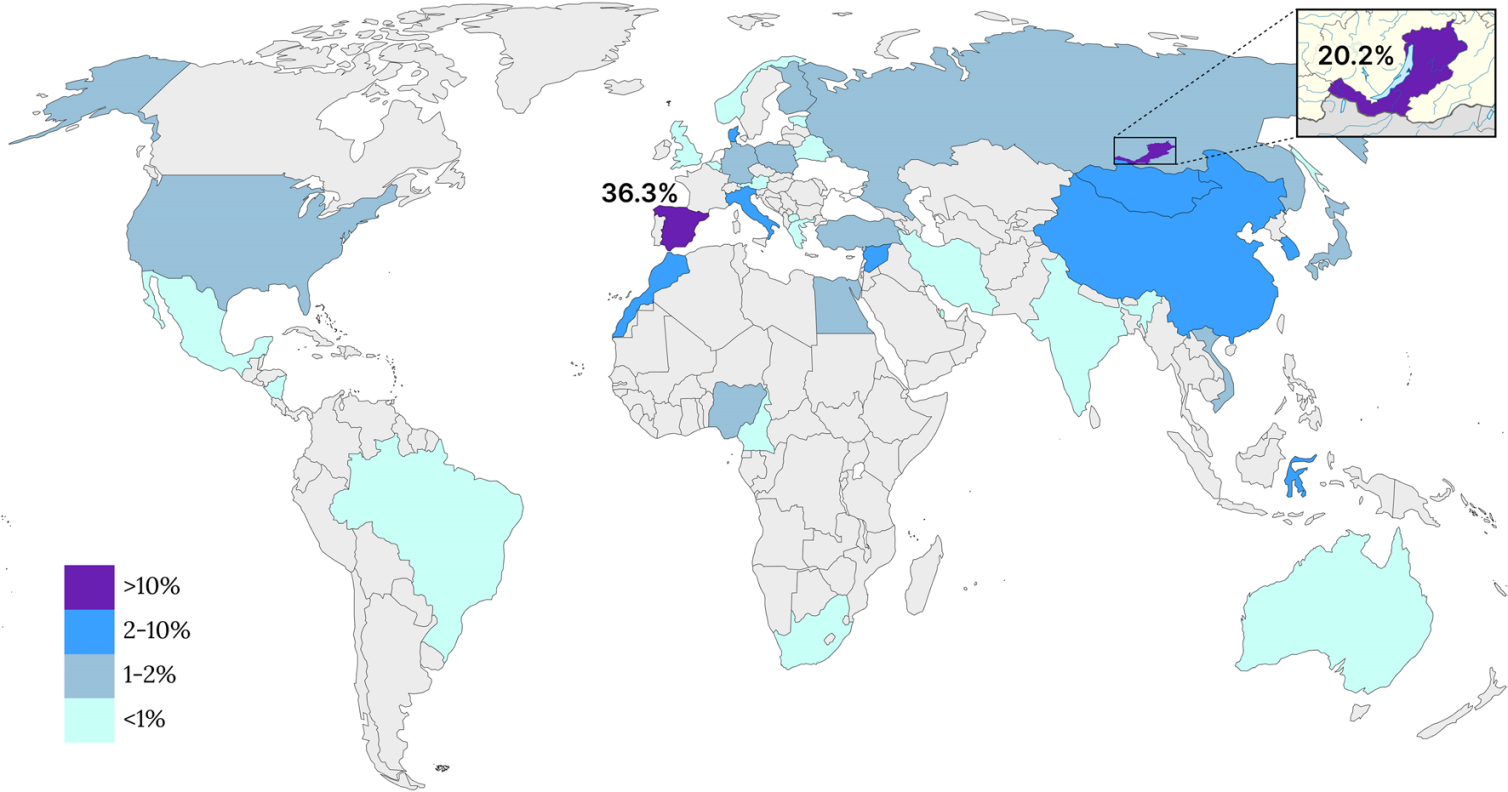
The worldwide prevalence of the m.1555A > G variant of the *MT-RNR1* gene among 47,328 patients with HL. *Note*: The full data is presented in the supplementary information (Table S2).

In order to update the global prevalence of the m.1555A > G variant, we conducted a systematic review of the literature, encompassing a total of 47,328 HL patients (Fig. 3, Table S2). Our analysis indicates that the global prevalence of the m.1555A > G variant in HL patients stands at approximately 1.8% (863/47,328). Notably, the m.1555A > G variant exhibits higher prevalence in Asia at 2.48% (701/28,271). In contrast, the variant is observed at average frequencies in Africa (1.01%; 5/493) and the Middle East (1.43%; 10/698). The Europe, America, and Australia report relatively lower prevalence’s of 0.97% (90/9,237), 0.92% (50/5,409), and 0.22% (7/3,220), respectively (Fig. 3, Table S2).

Some authors suggest that the relatively high prevalence of the m.1555A > G variant in Asia (2.48%) may be associated with a wider use of aminoglycoside antibiotics in countries of this region^25–27,29^. On the other hand, the relatively low prevalence of the m.1555A > G variant in Europe (0.97%) and America (0.92%) may be associated with underestimation of mitochondrial forms of HL (Table S2). As of now in the DNA-testing of the hereditary HL, including tests using NGS technologies, the pathogenic m.1555A > G variant of the *MT-RNR1* gene is not included in all research protocols^30^. In general, despite the relatively low global prevalence of the m.1555A > G variant (from 0.22% in Australia to 2.48% in Asia), it is found across all continents (Fig. 3). The wide distribution of the m.1555A > G variant is probably due to its de novo emergence in different regions of the world, as there are known sporadic cases of the m.1555A > G variant^31^.

The first studies based on restriction fragment length polymorphism (RFLP) and D-loop sequencing of mitochondrial DNA among carriers of the m.1555A > G variant did not found common polymorphic patterns among patients of Caucasian, Asian and African descend^32^. Also, no common patterns were found among 10 families (out from 13) in Japan carrying the m.1555A > G variant, demonstrating the absence of a single origin of this pathogenic variant within one population^29^. Subsequent studies describing variability of mtDNA haplogroups in Chinese, Korean and Japanese pedigrees with the m.1555A > G variant, detected various haplogroups, predominantly of East-Eurasian origin (A, B, C, D, F, G, M, K, N, R and Y)^28,29,33–38^. In the United Kingdom, Spain and Cuba maternal lines of pedigrees carrying the m.1555A > G variant were affiliated with mtDNA haplogroups of West-Eurasian origin (H, I, J, K, T, U and V)^9,39–41^. In families from India both East- and West-Eurasian haplogroups (M5a’d, U2e1 and T2) were found^42,43^. In families of African descent with the m.1555A > G variant haplogroups L0 and L1 were identified^32,33,44^. These findings persuasively suggest that the m.1555A > G variant may occurred sporadically in situ and may have multiplied through the evolution of the mitochondrial DNA^9,28,29,32–44^. The phylogenetic trees of mtDNAs among the m.1555A > G variant carriers around the world are presented in the Supplementary information (Fig. S2).

Despite to the probability of the independent origin of the m.1555A > G variant in the different regions of the world, the extremely high prevalence of this variant has been identified in Spain (36.3%, 1200/3302) (Fig. 3, Table S2)^45–49^. Increased frequencies of the m.1555A > G variant only in this region of Europe suggest other factors contributing to spread of this pathogenic variant. However, the phylogenetic analysis of the complete mtDNA variation demonstrates that the *MT-RNR1* gene is unlikely to be a hotspot region, because other known variants m.827A > G, m.961 T > C, and m.1005 T > C in this gene define sub-haplogroups, which are spread among Asians (B4b’d’e’j, A5b and F2) and Native Americans (B4b’d’e’j)^33^. In this case, the high proportion of the m.1555A > G variant in Spain, compared with other European countries, could possibly be related to exposure to exacerbating environmental factors and aminoglycoside treatment^39^. However, no significant differences in nutrition, living conditions and treatment methods were found between Spain and neighboring European countries^39^. Although different haplogroups have been identified among the m.1555A > G variant carriers in Spain, the majority of these carriers belonged to the haplogroup H (76%)^39^. The authors suggest that increased frequency of the m.1555A > G variant in patients with one haplogroup H (with three of the six studied haplotypes were specific for the m.1555A > G variant) in Spain may be due to a founder effect^39^. However, they emphasize, that haplogroup H is not specific to Spain, as this mitochondrial lineage is dominate in Europe, which does not exclude the possibility of an independent origin of the m.1555A > G variant on this major mitochondrial background^39^. Re-evaluation studies that further dissected mtDNA haplogroup H in Iberia confirmed that the previously reported overrepresentation of haplogroup H (38 from 50 individuals) among Spanish families affected by HL due to the m.1555A > G variant is primarily associated with sub-haplogroup H3 (15 from 38). This is believed to be the result of a significant, likely ancient, founder event associated with Franco-Cantabrian refuge area was the source of late-glacial expansions of hunter-gatherers that repopulated much of Central and Northern Europe approximately 15,000 years ago^50^.

The second region with high prevalence of the mitochondrial form of HL caused by the m.1555A > G variant of the *MT-RNR1* gene, was found by us in the Baikal Lake region of Russia, where the proportion of the m.1555A > G variant among Buryat patients (20.2%) was comparable with Spanish patients (36.3%) (Fig. 3). We found local accumulation of the identified mitochondrial form of HL (4.5 per 10,000 peoples) in one district of the Republic of Buryatia (Table 1). We hypothesize that this uneven prevalence among the endogamous Siberian population could point to a founder effect, similar to Spanish carriers of the m.1555A > G variant in Iberia. To support this hypothesis, we conducted a complete mitochondrial genome analysis and identified the mtDNA haplogroups in 15 unrelated families carrying the m.1555A > G variant. Among these 15 pedigrees with the m.1555A > G variant, different sub-haplogroups were identified, all of which are belonged to East-Eurasians haplogroups: A, D, and F^51–61^. Specifically, we observed an overrepresentation of a single maternal lineage cluster (86.7%), affiliated with sub-haplogroup A5b (13 out of 14 Buryat patients). The remaining 13.4% individuals belonged to sub-haplogroup D5a2a1 (1 out 14 Buryat patients) and sub-haplogroup F1a1d (1 out of 1 Russian patient) (Fig. 2). Notably, all identified mitochondrial lineages were specific and had not been previously found among carriers of the m.1555A > G variant^9,28,29,32–44^. Although the phylogenetically close subclades (A5, F1 and D5) have been found in Japanese^29,32,33^, Chinese^34,36^ and Korean families with the m.1555A > G variant^28^ (Fig. S2).

Moreover, we noted with great interest, that maternal subclades (A5b, A5b1, A5b1b, D5a2a1 and F1a1d) detected in this study belong to clusters that are more prevalent in Eastern Asia^57,58,61–69^, than in regions of Northern Asia^70–85^. The clearest example is the mitochondrial lineage affiliated with sub-haplogroup A5b, which was detected among 92.9% of Buryat patients with m.1555A > G, and was previously unobserved either in general Buryat population^83^ or in other Mongolic-speaking peoples^84^. While the exact distribution range of the A5b lineage is still being defined, in continental Asia this sub-haplogroup has been found with minor rates among Turkic-speaking populations of Altaians, Kazakhs and Uighurs^86,87^, the phylogenetic analysis of haplogroup A5 indicates greater diversity of its different subclades in the Japanese archipelago^56,61,68^. In total, these findings suggest predominantly non-autochthonous East-Asian origin of the mitochondrial background among the m.1555A > G variant carriers in the Baikal Lake region, aligning with data of the multivariable ancestral components in the maternal genetic landscape of this Eastern Siberian region^83,84^.

In general, this study confirms that 92.9% of Buryat families affected by HL due to the m.1555A > G variant share a common cluster consisting of several subclades (A5b, A5b1, and A5b1b) of the mitochondrial lineage affiliated with sub-haplogroup A5b. This finding suggests a single origin of this pathogenic variant from a common ancestor in the majority of detected cases in the Baikal Lake region of Russia.

## Methods

### Brief information about studied region

The Republic of Buryatia includes 21 districts and two cities (Ulan-Ude and Severobaikalsk) (https://egov-buryatia.ru, accessed on 15 September 2022), with an area of 351.3 thousand km^2^. This region of the Russian Federation borders with Mongolia. The population of the Republic of Buryatia is 978,600 people, with an average density of 2.78 people/km^2^. The major ethnic groups are Buryats (30.1%) and Russians (59.4%) (https://burstat.gks.ru/vpn2020, accessed on 25 January 2023). The Buryats are a Mongolic-speaking people and one of the largest indigenous groups of Siberia. Buryats share many customs with Mongols, including nomadic herding and using portable dwellings—yurts. The majority of the Buryat population lives in the Republic of Buryatia, Irkutsk Oblast’ and Zabaykalsky Krai of Russia. Buryats also live in the northeastern part of Mongolia and China (Inner Mongolia).

### Study sample

The DNA samples of 165 patients with HI from 160 unrelated families were collected in 2019. The majority of patients were of Buryat (47.8%; n = 79) and Russian ethnicity (46.1%; n = 76). Patients of other ethnicities accounted for 6.0% (n = 10). The males accounted for 41.2% (n = 68) and females—58.8% (n = 97). The average age was 50.7 ± 15.5 years.

### Clinical and audiological analysis

For each patient, a medical history was collected, including the information on previous illnesses, allergological history, injuries and/or surgeries, the use of ototoxic drugs and the exposure to industrial noise. The hearing thresholds were determined by pure-tone audiometry, using a clinical tonal audiometer “AA222” (“Interacoustics”, Middelfart, Denmark), according to the current clinical standards. Air-conduction and bone conduction thresholds were obtained at 0.125, 0.25, 0.5, 1, 2, 4 and 8 kHz. Severity of hearing loss was defined by pure tone average (PTA_0.5,1,2,4 kHz_), as mild (25–40 dB), moderate (41–70 dB), severe (71–90 dB) or profound (above 90 dB).

### Detection of the m.1555A > G variant in the *MT-RNR1* gene

DNA was extracted using the phenol–chloroform method from the blood leukocytes. Detection of the m.1555A > G variant in the *MT-RNR1* gene was performed by PCR–RFLP analysis using the previously described oligonucleotide primer, and restriction ferment *Hae*III^9^. The presence of the m.1555A > G variant in the *MT-RNR1* gene was verified by Sanger sequencing using the original sequence of oligonucleotide primers: F—AAACGCTTAGCCTAGCCACA, R—GCTACACTCTGGTTCGTCCA, selected using the Primer-BLAST program^88^.

### Analysis of mtDNA haplogroups

Sequencing of the mitochondrial genome by next generation sequencing (NGS) was performed using Illumina NextSeq 500. Haplogroups were determined in accordance with PhyloTree.org mtDNA nomenclature—mtDNA tree Build 17^89^.

### Ethics approval and consent to participate

All methods were performed in accordance with relevant guidelines and regulations. Written informed consent was obtained from all patients participating in the study. The study was conducted according to the guidelines of the Declaration of Helsinki and approved by the local Biomedical Ethics Committee at the Yakut Scientific Center of Complex Medical Problems, Yakutsk, Russia (Yakutsk, protocol No. 50 of 24 December 2019).

## Conclusion

In this study we found the high prevalence of m.1555A > G variant of the *MT-RNR1* gene among patients with HL residing in Baikal Lake region of Russia. This establishes Eastern Siberia as region with the second most extensive accumulation of mitochondrial form of HL in the world after South Europe. Analysis of the complete mitochondrial genome in 14 unrelated Buryat families carrying the m.1555A > G variant revealed common mitochondrial background affiliated with sub-haplogroup A5b (92.9%), which was not previously reported in the Eastern Siberia. Considering that over 90% of Buryat families affected by HL due to the m.1555A > G variant belong to one maternal lineage, we conclude that the high prevalence of this pathogenic variant in the Baikal Lake region is likely due to a founder effect. These findings expand our knowledge of the impact of the population bottleneck effects on the genetic epidemiology of the mitochondrial form of HL caused by the m.1555A > G variant of the *MT-RNR1* gene.

### Supplementary Information

Supplementary Information 1.

Supplementary Information 2.

Supplementary Information 3.

Supplementary Information 4.

## Data Availability

The raw datasets analyzed during the study are available from the corresponding author on reasonable request.
