# Supplementary material for: High prevalence of m.1555A > G in patients with hearing loss in the Baikal Lake region of Russia as a result of founder effect
Source: Sci Rep. 2024 Jul 3;14:15342. doi: 10.1038/s41598-024-66254-z (PMC11222474; doi:10.1038/s41598-024-66254-z)
Supplement: Supplementary file 1 — Supplementary Information 1. [file 41598_2024_66254_MOESM1_ESM.docx]

**Supplementary legend**

Figure S1. Original electrophoregram;

Figure S2. The phylogenetic trees of mtDNAs among m.1555A>G carriers around the world;

Table S1. Clinical characteristics of the 21 patients with hearing loss due to the m.1555A>G in the *MT-RNR1* gene in Republic of Buryatia (Eastern Siberia, Russia);

Table S2. The prevalence of the m.1555A>G variant in the *MT-RNR1* gene among 47,328 patients with hearing loss around the world.
