# Supplementary material for: High prevalence of m.1555A > G in patients with hearing loss in the Baikal Lake region of Russia as a result of founder effect
Source: Sci Rep. 2024 Jul 3;14:15342. doi: 10.1038/s41598-024-66254-z (PMC11222474; doi:10.1038/s41598-024-66254-z)
Supplement: Supplementary file 2 — Supplementary Information 2. [file 41598_2024_66254_MOESM2_ESM.docx]

**Supplementary information**

**Table S1**. Clinical characteristics of the 21 patients with hearing loss due to the m.1555A>G in the *MT-RNR1* gene in Republic of Buryatia (Eastern Siberia, Russia)

| **Number of family** | **Sex** | **Ethnicity** | **Age**  **(in years)** | **Degree of HL** | **Age of HL onset (in years)** | **History of aminoglycoside use** | **mtDNA haplogroup** |
| --- | --- | --- | --- | --- | --- | --- | --- |
| 5 | M | Buryat | 50 | Profound | 0 | - | A5b |
| 1 | F | Buryat | 57 | Severe | 2 | - | A5b |
| 6 | F | Buryat | 64 | Profound | 9 | - | A5b |
| 2 | F | Buryat | 59 | Profound (right ear)  Severe (left ear) | 3 | - | A5b1 |
| 7 | M | Buryat | 59 | Profound | 10 | - | A5b |
| 3 | F | Buryat | 51 | Profound | early childhood | - | A5b |
| 8 | F | Buryat | 57 | Profound | early childhood | - | A5b |
| 9 | F | Buryat | 56 | Profound | 0 | - | A5b |
| 4 | M | Buryat | 59 | Profound | 2 | - | A5b |
| 1 | F | Buryat | 51 | Profound | 0 | - | - |
| 10 | M | Buryat | 45 | Profound | 3 | + | D5a2a1 |
| 11 | F | Buryat | 47 | Profound | 0 | - | A5b1b |
| 12 | F | Buryat | 70 | Profound | 11 | + | A5b |
| 13 | M | Buryat | 54 | Profound | 0 | - | A5b |
| 14 | F | Russian | 50 | Profound (right ear)  Severe (left ear) | 2 | - | F1a1d |
| 15 | M | Buryat | 43 | Profound | 3 | + | A5b |
| 2 | M | Buryat | 53 | Profound | 0 | - | - |
| 2 | M | Buryat | 38 | Profound | 0 | - | - |
| 2 | F | Buryat | 53 | Profound | 0 | - | A5b |
| 2 | F | Buryat | 59 | Profound | 7 | + | - |
| 2 | M | Buryat | 43 | Profound | 4 months | + | - |

**Note**: F – female; M – Male; HL – hearing loss; (+) - Positive history of aminoglycoside use; (-) - Negative history of aminoglycoside use.

**.**

**Table S2.** The prevalence of the m.1555A>G variant in the *MT-RNR1* gene among 47,328 patients with hearing loss around the world.

| **Region** | | **Country** | **Prevalence** | **Number of patients with m.1555A>G** | **Sample**  **(region)** | **Reference** |
| --- | --- | --- | --- | --- | --- | --- |
| America | North America | USA | 0.60% | 1 | 164 | [1] |
|  |  |  | 0.73% | 18 | 2434 | [2] |
|  |  |  | 0% | 0 | 109 | [3] |
|  |  |  | 5% | 7 | 459 | [4] |
|  |  |  | 0.92% | 2 | 217  (Florida) | [5] |
|  |  |  | 17% | 7 | 41  (Los Angeles) | [6] |
|  |  | Total | 1.02% | 35 | 3424 |  |
|  |  | Mexico | 0% | 0 | 33 | [7] |
|  |  |  | 0% | 0 | 76 | [8] |
|  |  | Total | 0% | 0 | 109 |  |
|  | Central and South America | Brazil | 0.60% | 3 | 542 | [9] |
|  |  |  | 2% | 4 | 403 | [10] |
|  |  |  | 0.46% | 3 | 645 | [11] |
|  |  |  | 0% | 0 | 27 | [12] |
|  |  |  | 1.20% | 1 | 78 | [13] |
|  |  |  | 2.60% | 4 | 152 | [14] |
|  |  | Total | 0.81% | 15 | 1847 |  |
|  |  | Nicaragua | 0% | 0 | 29 | [15] |
| **Total (America)** | | | **0.92%** | **50** | **5409** |  |
| Europe | North Europe | Norway | 0% | 0 | 32 | [16] |
|  |  | Finland | 0.97% | 1 | 103  (North of Finland) | [17] |
|  |  |  | 2.56% | 3 | 117 | [18] |
|  |  |  | 0% | 0 | 227 | [19] |
|  |  | Denmark | 2.40% | 2 | 85 | [20] |
|  |  | Total | 1.06% | 6 | 564 |  |
|  | Central and Eastern Europe | United Kingdom | 0% | 0 | 202 | [21] |
|  |  |  | 0.56% | 12 | 2147 | [22] |
|  |  |  | 2.5% | 2 | 80 | [19] |
|  |  | Total | 0.58% | 14 | 2429 |  |
|  |  | Germany | 0.63% | 1 | 160 | [23] |
|  |  |  | 2.40% | 3 | 125 |  |
|  |  |  | 0% | 0 | 56 |  |
|  |  | Total | 1.17% | 4 | 341 |  |
|  |  | Belgium | 0.34% | 2 | 588 | [24] |
|  |  | Austria | 0% | 0 | 77 | [25] |
|  |  | Estonia | 0.43% | 1 | 233 | [26] |
|  |  | Poland | 2.40% | 3 | 125 | [23] |
|  |  |  | 1.30% | 20 | 1499 | [27] |
|  |  |  | 3.60% | 9 | 250 | [28] |
|  |  | Total | 1.7% | 32 | 1874 |  |
|  |  | Belarus | 0.51% | 2 | 391 | [29] |
|  |  | Russia | 0.82% | 1 | 122  (St. Petersburg - Russians) | [30] |
|  |  |  | 0% | 0 | 135  (Volga-Ural region - Russians, Tatars, Bashkirs) |  |
|  |  |  | 0% | 0 | 88  (Republic of Altai - Altaians) |  |
|  |  |  | 1.54% | 1 | 65  (Republic of Sakha (Yakutia) - Yakuts) |  |
|  |  |  | 3.92% | 4 | 102  (St. Petersburg) | [31] |
|  |  |  | 1.67% | 1 | 60  (Republic of Sakha (Yakutia) - Yakuts) | [32] |
|  |  |  | 0% | 0 | 20  (Republic of Sakha (Yakutia) - Russians) |  |
|  |  |  | 0% | 0 | 220  (Republic of Tuva - Tuvinians) | [33] |
|  |  |  | 0% | 0 | 93  (Republic of Altai - Altaians) |  |
|  |  |  | 17.39% | 4 | 23  (Republic of Sakha (Yakutia) - Evens) | [34] |
|  |  |  | **20.27%** | **15** | **74**  **(Republic of Buryatia - Buryats)** | **This study*** |
|  |  |  | **1.33%** | **1** | **75**  **(Republic of Buryatia – Russians)** |  |
|  |  | Total | 1.18% | 11 | 928 |  |
|  | South Europe | Italy | 1.56% | 2 | 128  (Southern Italy) | [19] |
|  |  |  | 1.70% | 3 | 169 | [35] |
|  |  |  | 0% | 0 | 125 | [36] |
|  |  |  | 5.30% | 9 | 167 | [37] |
|  |  |  | 0% | 0 | 102 | [38] |
|  |  | Total | 2.03% | 14 | 691 |  |
|  |  | **Spain*** | **17%** | **9** | **54** | **[39]** |
|  |  |  | **20%** | **42** | **209** | **[40]** |
|  |  |  | **23.70%** | **9** | **38** | **[41]** |
|  |  |  | **28.57%** | **6** | **21** | **[42]** |
|  |  |  | **0.91%** | **2** | **219** | **[43]** |
|  |  |  | **41%** | **1132** | **2761** | **[44]** |
|  |  | **Total** | **36.34%** | **1200** | **3302** |  |
|  |  | North Macedonia | 0% | 0 | 130 | [45] |
|  |  | Greece | 0.41% | 2 | 478 | [46] |
|  |  |  | 0.42% | 2 | 513 | [47] |
|  |  | Total | 0.4% | 4 | 991 |  |
| **Total (Europe)** | | | **0.97%** | **90** | **9237** |  |
| Asia | East Asia | Mongolia | 1.06% | 2 | 188 | [48] |
|  |  |  | 7.70% | 31 | 480 | [49] |
|  |  | Total | 4.94% | 33 | 668 |  |
|  |  | China | 1.25% | 15 | 1201  (Shanxi province) | [50] |
|  |  |  | 3.96% | 65 | 1642  (Zhejiang Province) | [51] |
|  |  |  | 4.40% | 5 | 114  (Shanghai) | [52] |
|  |  |  | 10.17% | 6 | 59  (Chongqing, Yongchuan District) | [53] |
|  |  |  | 3.23% | 19 | 588 | [54] |
|  |  |  | 4.96% | 6 | 121  (Northwest China - Tibetans) | [55] |
|  |  |  | 0.83% | 5 | 49  (Northwest China - Tu) |  |
|  |  |  | 10.20% | 1 | 19  (Northwest China - Mongolians) |  |
|  |  |  | 8.60% | 8 | 93 | [56] |
|  |  |  | 5.21% | 126 | 2417  (Northwest China) | [57] |
|  |  |  | 8.40% | 67 | 802  (Gansu Province) | [58] |
|  |  |  | 1.09% | 1 | 92 | [59] |
|  |  |  | 1.32% | 2 | 152  (Zhengzhou City, Henan Province) | [60] |
|  |  |  | 1.26% | 5 | 398 | [61] |
|  |  |  | 9.52% | 8 | 84  (Southwest China) | [62] |
|  |  |  | 0.23% | 2 | 879 | [63] |
|  |  |  | 1.97% | 102 | 5184  (Taiwan) | [64] |
|  |  |  | 6.82% | 30 | 440 | [65] |
|  |  | Total | 3.30% | 473 | 14334 |  |
|  |  | Japan | 3.45% | 11 | 319 | [66] |
|  |  |  | 10% | 14 | 140 |  |
|  |  |  | 5.07% | 7 | 138 | [67] |
|  |  |  | 2.07% | 3 | 145 | [68] |
|  |  |  | 1.30% | 129 | 10047 | [69] |
|  |  | Total | 1.52% | 164 | 10789 |  |
|  |  | South Korea | 0.88% | 2 | 227 | [70] |
|  |  |  | 3.20% | 9 | 281 | [71] |
|  |  | Total | 2.17% | 11 | 508 |  |
|  | South-East Asia | Indonesia | 5.30% | 4 | 75  (Sulawesi) | [72] |
|  |  | Vietnam | 1.15% | 1 | 87 | [73] |
|  | South Asia | India | 1% | 3 | 303  (Hyderabad) | [74] |
|  |  |  | 0.68% | 5 | 729  (South India) | [75] |
|  |  |  | 1.14% | 7 | 616 | [76] |
|  |  | Total | 1.1% | 20 | 1810 |  |
| **Total (Asia)** | | | **2.48%** | **701** | **28271** |  |
| Middle East | | Syria | 4.45 | 6 | 132 | [77] |
|  |  | Qatar | 0% | 0 | 126 | [78] |
|  |  | Turkey | 0% | 0 | 95 | [79] |
|  |  |  | 1.80% | 3 | 168 | [80] |
|  |  |  | 1.40% | 1 | 70 | [81] |
|  |  | Total | 1.20% | 4 | 333 |  |
|  |  | Iran | 0% | 0 | 107 | [82] |
| **Total (Middle East)** | | | **1.43%** | **10** | **698** |  |
| Africa | North Africa | Egypt | 1.30% | 1 | 97 | [83] |
|  |  | Morocco | 3.60% | 3 | 84 | [84] |
|  | Sub-Saharan Africa | Cameroon | 0% | 0 | 70  (North Cameroon) | [85] |
|  |  | Nigeria | 1.67% | 1 | 60 | [86] |
|  |  | South Africa | 0% | 0 | 182 | [87] |
| **Total (Africa)** | | | **1.01%** | **5** | **493** |  |
| Australia | | Australia | 0.27% | 1 | 364 | [88] |
|  |  |  | 0.21% | 6 | 2856 | [89] |
| **Total (Australia)** | | | **0.22%** | **7** | **3220** |  |
| **Total (Worldwide)** | | | **1.82%** | **863** | **47,328** | |

**Note**: Regions with a >20% prevalence were not included in the calculation (highlighted in bold).

**References**

1. Li, R., Greinwald, J.H., Yang, L., Choo, D.I., Wenstrup, R.J., Guan, M.-X. Molecular Analysis of the Mitochondrial 12S RRNA and tRNASer(UCN) Genes in Paediatric Subjects with Non-Syndromic Hearing Loss. *J Med Genet*, **41**, 615–620, doi:10.1136/jmg.2004.020230. (2004)
2. Yelverton, J.C., Arnos, K., Xia, X.-J., Nance, W.E., Pandya, A., Dodson, K.M. The Clinical and Audiologic Features of Hearing Loss Due to Mitochondrial Mutations. *Otolaryngol Head Neck Surg*. **148**, 1017–1022, doi:10.1177/0194599813482705. (2013)
3. Samanich, J., Lowes, C., Burk, R., Shanske, S., Lu, J., Shanske, A. *et al.* Mutations in GJB2, GJB6, and Mitochondrial DNA Are Rare in African American and Caribbean Hispanic Individuals with Hearing Impairment. *Am J Med Genet A.* **143A**, 830–838, doi:10.1002/ajmg.a.31668. (2007)
4. Seligman, K.L., Shearer, A.E., Frees, K., Nishimura, C., Kolbe, D., Dunn, C. *et al*. Genetic Causes of Hearing Loss in a Large Cohort of Cochlear Implant Recipients. *Otolaryngol Head Neck Surg.* **166**, 734–737, doi:10.1177/01945998211021308. (2022)
5. Vivero, R.J., Ouyang, X., Yan, D., Du, L., Liu, W., Angeli, S.I. *et al.* Mitochondrial DNA Mutation Screening in an Ethnically Diverse Nonsyndromic Deafness Cohort*. Genet Test Mol Biomarkers.* **16**, 1146–1148, doi:10.1089/gtmb.2011.0365. (2012)
6. Fischel-Ghodsian, N., Prezant, T.R., Chaltraw, W.E., Wendt, K.A., Nelson, R.A., Arnos, K.S. *et al.* Mitochondrial Gene Mutation Is a Significant Predisposing Factor in Aminoglycoside Ototoxicity. *Am J Otolaryngol*. **18**, 173–178, doi:10.1016/s0196-0709(97)90078-8. (1997)
7. Meza, G., Torres-Ruíz, N.M., Tirado-Gutiérrez, C., Aguilera, P. MmtDNA Mutations, Hearing Loss and Aminoglycoside Treatment in Mexicans. *Braz J Otorhinolaryngol*. **77**, 573–576, doi:10.1590/s1808-86942011000500006. (2011)
8. de la Luz Arenas-Sordo, M., Menendez, I., Hernández-Zamora, E., Sirmaci, A., Gutiérrez-Tinajero, D., McGetrick, M. *et al*. Unique Spectrum of GJB2 Mutations in Mexico. *Int J Pediatr Otorhinolaryngol.* **76**, 1678–1680, doi:10.1016/j.ijporl.2012.08.005. (2012)
9. Batissoco, A.C., Pedroso-Campos, V., Pardono, E., Sampaio-Silva, J., Sonoda, C.Y., Vieira-Silva, G.A. *et al.* Molecular and Genetic Characterization of a Large Brazilian Cohort Presenting Hearing Loss. *Hum Genet.* **141**, 519–538, doi:10.1007/s00439-021-02372-2. (2022)
10. Abreu-Silva, R.S., Lezirovitz, K., Braga, M.C.C., Spinelli, M., Pirana, S., Della-Rosa, V.A. *et al.* Prevalence of the A1555G (12S rRNA) and tRNASer(UCN) Mitochondrial Mutations in Hear-ing-Impaired Brazilian Patients. *Braz J Med Biol Res*. **39**, 219–226, doi:10.1590/s0100-879x2006000200008. (2006)
11. de Oliveira, C.A., Alexandrino, F., Christiani, T.V., Steiner, C.E., Cunha, J.L.R., Guerra, A.T.M. *et al.* Molecular Genetics Study of Deafness in Brazil: 8-Year Experience. *Am J Med Genet A*. **143A**, 1574–1579, doi:10.1002/ajmg.a.31838. (2007)
12. Maniglia, L.P., Moreira, B.C.L., da Silva, M.A.O.M., Piatto, V.B., Maniglia, J.V. Screening of the Mitochondrial A1555G Mutation in Patients with Sensorineural Hearing Loss. *Braz J Otorhinolaryngol*. **74**, 731–736, doi:10.1016/S1808-8694(15)31384-7. (2008)
13. Salomão, K.B., Ayo, C.M., Della-Rosa, V.A. Investigation of the A1555G Mutation in Mitochondrial DNA (MT-RNR1) in Groups of Brazilian Individuals with Nonsyndromic Deafness and Normal-Hearing. *Indian J Hum Genet.* **19**, 54–57, doi:10.4103/0971-6866.112888. (2013)
14. Alves, R.M., da Silva Costa, S.M., do Amôr Divino Miranda, P.M., Ramos, P.Z., Marconi, T.G., Santos Oliveira, G. *et al.* Analysis of Mitochondrial Alterations in Brazilian Patients with Sensorineural Hearing Loss Using MALDI-TOF Mass Spectrometry. *BMC Med Genet*, **17**, 41, doi:10.1186/s12881-016-0303-5. (2016)
15. Saunders, J.E., Greinwald, J.H., Vaz, S., Guo, Y. Aminoglycoside Ototoxicity in Nicaraguan Children: Patient Risk Factors and Mitochondrial DNA Results. *Otolaryngol Head Neck Surg*. **140**, 103–107, doi:10.1016/j.otohns.2008.09.027. (2009)
16. Siem, G., Fagerheim, T., Jonsrud, C., Laurent, C., Teig, E., Harris, S. *et al.* Causes of Hearing Impairment in the Norwegian Paediatric Cochlear Implant Program. *Int J Audiol*. **49**, 596–605, doi:10.3109/14992021003743269. (2010)
17. Häkli, S., Luotonen, M., Sorri, M., Majamaa, K. Mutations in the Two Ribosomal RNA Genes in Mitochondrial DNA among Finnish Children with Hearing Impairment*. BMC Med Genet.* 16**,** 3, doi:10.1186/s12881-015-0145-6. (2015)
18. Lehtonen, M.S., Uimonen, S., Hassinen, I.E., Majamaa, K. Frequency of mitochondrial DNA point mutations among patients with familial sensorineural hearing impairment. *Eur J Hum Genet*. **8**, 315-8. doi: 10.1038/sj.ejhg.5200455. (2000)
19. Jacobs, H.T., Hutchin, T.P., Käppi, T., Gillies, G., Minkkinen, K., Walker, J. *et al.* Mitochondrial DNA mutations in patients with postlingual, nonsyndromic hearing impairment. *Eur J Hum Genet*. **13**, 26-33. doi: 10.1038/sj.ejhg.5201250. (2005)
20. ØStergaard, E., Montserrat-Sentis, B., Grønskov, K., Brøndum-Nielsen, K. The A1555G MtDNA Mutation in Danish Hearing-Impaired Patients: Frequency and Clinical Signs. *Clin Genet*, **62**, 303–305, doi:10.1034/j.1399-0004.2002.620408.x. (2002)
21. Hutchin, T.P., Lench, N.J., Arbuzova, S., Markham, A.F., Mueller, R.F. Maternally Inherited Hearing Impairment in a Family with the Mitochondrial DNA A7445G Mutation. *Eur J Hum Genet*. **9**, 56–58, doi:10.1038/sj.ejhg.5200581. (2001)
22. Kullar P., Alston, C.L., Ball, S., Blakely, E.L., Differ, A.M., Fratter, C. *et al.* The Frequency of the m.1555A>G (MT-RNR1) Variant in UK Patients with Suspected Mitochondrial Deafness. *Hearing Balance Commun*. **14**, 101–102, doi:10.3109/21695717.2016.1151124. (2016)
23. Kupka, S., Tóth, T., Wróbel, M., Zeissler, U., Szyfter, W., Szyfter, K. *et al*. Mutation A1555G in the 12S rRNA Gene and Its Epidemiological Importance in German, Hungarian, and Polish Patients. *Hum Mutat*. **19**, 308–309, doi:10.1002/humu.9017. (2002)
24. Konings, A., Van Camp, G., Goethals, A., Van Eyken, E., Vandevelde, A., Ben Azza, J. *et al.* Mutation Analysis of Mitochondrial DNA 12SrRNA and tRNASer(UCN) Genes in Non-Syndromic Hearing Loss Patients. *Mitochondrion*,. **8**, 377–382, doi:10.1016/j.mito.2008.08.001. (2008)
25. Ramsebner, R., Lucas, T., Schoefer, C., Ludwig, M., Baumgartner, W.-D., Wachtler, F.J. *et al.* Relevance of the A1555G Mutation in the 12S rRNA Gene for Hearing Impairment in Austria. *Otol Neurotol*, **28**, 884–886, doi:10.1097/MAO.0b013e3181461b26. (2007)
26. Teek, R., Kruustük, K., Žordania, R., Joost, K., Kahre, T., Tõnisson, N. *et al*. Hearing Impairment in Estonia: An Algorithm to Investigate Genetic Causes in Pediatric Patients. *Adv Med S*ci, **58**, 419–428, doi:10.2478/ams-2013-0001. (2013)
27. Pollak, A., Lechowicz, U., Kędra, A., Stawiński, P., Rydzanicz, M., Furmanek, M. *et al*. Novel and De Novo Mutations Extend Association of POU3F4 with Distinct Clinical and Radiological Phenotype of Hearing Loss. *PLoS One*. **11**, e0166618, doi:10.1371/journal.pone.0166618. (2016)
28. Rydzanicz, M., Wróbel, M., Pollak, A., Gawecki, W., Brauze, D., Kostrzewska-Poczekaj, M. *et al*. Mutation Analysis of Mitochondrial 12S RRNA Gene in Polish Patients with Non-Syndromic and Aminoglycoside-Induced Hearing Loss. *Biochem Biophys Res Commun*. **395**, 116–121, doi:10.1016/j.bbrc.2010.03.149. (2010)
29. Danilenko, N. *et al.* Spectrum of genetic changes in patients with non-syndromic hearing impairment and extremely high carrier frequency of 35delG GJB2 mutation in Belarus. *PLoS One.,* 7, e36354. doi: 10.1371/journal.pone.0036354. (2012)
30. Dzhemileva, L.U., Posukh, O.L., Tazetdinov, A.M., Barashkov, N.A., Zhuravskiĭ, S.G., Ponidelko, S.N. *et al.* [Analysis of mitochondrial 12S rRNA and tRNA(Ser(UCN)) genes in patients with nonsyndromic sensorineural hearing loss from various regions of Russia]. *Genetika.* **45**, 982–991. (2009)
31. Zhuravsky, S.G. Sensorineural hearing loss: molecular genetics, structural and therapeutic and preventive aspects (clinical and experimental study) [Russian: Zhuravskiy S.G. Sensonevral'naya tugoukhost': mole-kulyarno-geneticheskiye, strukturnyye i lechebno-profilakticheskiye aspekty (kliniko-eksperimental'noye issle-dovaniye]. Doctoral thesis. Pavlov First State Medical University of St. Petersburg, Russian Federation, St. Peters-burg. (2006)
32. Romanov, G.P., Barashkov, N.A., Teryutin, F.M. Frequency of m.1555A>G mutation in MT-RNR1 gene of mitochondrial DNA among deaf individuals in Yakutia. *Yakut Medical Journal*, **59**, 49–51. (2017)
33. Danilchenko, V.Y. Analysis of genetic control of hereditary hearing loss in populations a number of regions of Si-beria [Russian: Danil'chenko V.Y. Analiz geneticheskogo kontrolya nasledstvennoy poteri slukha v pop-ulyatsiyakh ryada regionov Sibiri]. PhD thesis. Institute of Cytology and Genetics, Siberian Branch of Russian Academy of Sciences, Russian Federation, Novosibirsk. (2022)
34. Pshennikova, V.G., Teryutin, F.M., Romanov, G.P, Solovyov, A.V., Barashkov, N.A. A local focus of accumulation of the mitochondrial form of hearing loss in Even-Bytantaysky district of Yakutia. *Yakut Medical Journal.* **80**, 86–90, doi:10.25789/YMJ.2022.79.19. (2022)
35. Guaran, V., Astolfi, L., Castiglione, A., Simoni, E., Olivetto, E., Galasso, M. *et al.* Association between Idiopathic Hearing Loss and Mitochondrial DNA Mutations: A Study on 169 Hearing-Impaired Subjects. *Int J Mol Med*. **32**, 785–794, doi:10.3892/ijmm.2013.1470. (2013)
36. Morgan, A., Lenarduzzi, S., Spedicati, B., Cattaruzzi, E., Murru, F.M., Pelliccione, G. *et al.* Lights and Shadows in the Genetics of Syndromic and Non-Syndromic Hearing Loss in the Italian Population. *Genes (Basel).* **11**, 1237, doi:10.3390/genes11111237. (2020)
37. Berrettini, S., Forli, F., Passetti, S., Rocchi, A., Pollina, L., Cecchetti, D. *et al.* Mitochondrial Non-Syndromic Sensorineural Hearing Loss: A Clinical, Audiological and Pathological Study from Italy, and Re-vision of the Literature. *Biosci Rep*. **28**, 49–59, doi:10.1042/BSR20070027. (2008)
38. Spedicati, B., Santin, A., Nardone, G.G., Rubinato, E., Lenarduzzi, S., Graziano, C. *et al.* The Enigmatic Genetic Landscape of Hereditary Hearing Loss: A Multistep Diagnostic Strategy in the Italian Population. *Biomedicines.* **11**, 703, doi:10.3390/biomedicines11030703. (2023)
39. Bravo, O., Ballana, E., Estivill, X. Cochlear Alterations in Deaf and Unaffected Subjects Carrying the Deaf-ness-Associated A1555G Mutation in the Mitochondrial 12S rRNA Gene. *Biochem Biophys Res Commun*. **344**, 511–516, doi:10.1016/j.bbrc.2006.03.143. (2006)
40. López-Bigas, N., Rabionet, R., Martinez, E., Bravo, O., Girons, J., Borragan, A. *et al*. Mutations in the Mitochondrial tRNA Ser(UCN) and in the GJB2 (Connexin 26) Gene Are Not Modifiers of the Age at Onset or Severity of Hearing Loss in Spanish Patients with the 12S RRNA A1555G Mutation. *Am J Hum Genet*. **66**, 1465–1467, doi:10.1086/302870. (2000)
41. Gallo-Terán, J., Arellano, B., Morales-Angulo, C., Modamio-Høybjør, S., Moreno-Pelayo, M.Á., Ramírez-Camacho, R. *et al.* Prevalencia de la mutación a1555g en el adn mitocondrial en pacientes con patología auditiva o vestibular debida a la ototoxicidad de los aminoglucósidos. *Acta Otorrinolaringológica Española.* **55**, 212–217, doi:10.1016/S0001-6519(04)78511-8. (2004)
42. Gallo-Terán, J., Morales-Angulo, C., del Castillo, I., Villamar, M., Moreno-Pelayo, M., García-Mantilla, J. *et al.* Incidencias de Las Mutaciones A1555G en el ADN Mitocondrial y 35delG en el Gen GJB2 (Conexina 26) En Fa-milias Con Hipoacusia Neurosensorial Postlocutiva No Sindrómica En Cantabria. *Acta Otorrinolaringológica Española*. **53**, doi:10.1016/S0001-6519(02)78349-0. (2002)
43. Morales Angulo, C., Gallo-Terán, J., Señaris, B., Fontalva, A., González-Aguado, R., Fernández-Luna, J.L. Prevalencia de la mutación A1555G del gen MT-RNR1 en pacientes con hipoacusia postlocutiva sin antecedentes familiares de sordera. *Acta otorrinolaringol. esp*, 83–86. (2011)
44. Bellusci, M., Paredes-Fuentes, A.J., Ruiz-Pesini, E., Gómez, B., MITOSPAIN Working Group, Martín, M.A. *et al.* The Genetic Landscape of Mitochondrial Diseases in Spain: A Nationwide Call*. Genes (Basel).* **12**, 1590, doi:10.3390/genes12101590. (2021)
45. Sukarova Stefanovska, E., Cakar, M., Filipce, I., Plaseska Karanfilska, D. Genetics of Non Syndromic Hearing Loss in the Republic of Macedonia. *Balkan J Med Genet*, **15**, 57–59, doi:10.2478/v10034-012-0020-0. (2012)
46. Kokotas, H., Grigoriadou, M., Korres, G.S., Ferekidou, E., Papadopoulou, E., Neou, P. *et al.* The A1555G Mitochondrial DNA Mutation in Greek Patients with Non-Syndromic, Sensorineural Hearing Loss. *Biochem Biophys Res Commun.* **390**, 755–757, doi:10.1016/j.bbrc.2009.10.044. (2009)
47. Kokotas, H., Grigoriadou, M., Korres, G.S., Ferekidou, E., Kandiloros, D., Korres, S. *et al.* Detection of Deafness-Causing Mutations in the Greek Mitochondrial Genome. *Dis Markers.* **30**, 283–289, doi:10.3233/DMA-2011-0786. (2011)
48. Erdenechuluun, J., Lin, Y.-H., Ganbat, K., Bataakhuu, D., Makhbal, Z., Tsai, C.-Y. *et al.* Unique Spectra of Deafness-Associated Mutations in Mongolians Provide Insights into the Genet-ic Relationships among Eurasian Populations. *PLoS One*. **13**, e0209797, doi:10.1371/journal.pone.0209797. (2018)
49. Pandya, A., Xia, X.J., Erdenetungalag, R., Amendola, M., Landa, B., Radnaabazar, J., Dangaasuren, B. *et al.* Heterogenous Point Mutations in the Mitochondrial tRNA Ser(UCN) Precursor Coexisting with the A1555G Mutation in Deaf Students from Mongolia. A*m J Hum Genet*. **65**, 1803–1806, doi:10.1086/302658. (1999)
50. Zhou, Y. *et al*. Mutation Analysis of Common Deafness Genes among 1,201 Patients with Non-Syndromic Hearing Loss in Shanxi Province. *Mol Genet Genomic Med*, **7**, e537, doi:10.1002/mgg3.537. (2019)
51. Lu, J., Li, Z., Zhu, Y., Yang, A., Li, R., Zheng, J. *et al*. Mitochondrial 12S rRNA Variants in 1642 Han Chinese Pediatric Subjects with Aminoglycoside-Induced and Nonsyndromic Hearing Loss. *Mitochondrion*. **10**, 380–390, doi:10.1016/j.mito.2010.01.007. (2010)
52. Chai, Y., Pang, X., Chen, D., Li, L., Chen, Y., Sun, L. *et al.* Molecular Etiology of Non-Dominant, Non-Syndromic, Mild-to-Moderate Childhood Hearing Impairment in Chinese Hans. *Am J Med Genet A*, **164A**, 3115–3119, doi:10.1002/ajmg.a.36785. (2014)
53. Jiang, H., Liu, Q., Chen, L. Screening and Analysis of Mutation Hot-Spots in Deafness-Associated Genes among Adolescents with Hearing Loss. *Mol Med Rep.* **12**, 8179–8184, doi:10.3892/mmr.2015.4475. (2015)
54. Wu, J., Cao, Z., Su, Y., Wang, Y., Cai, R., Chen, J. *et al*. Molecular Diagnose of a Large Hearing Loss Population from China by Targeted Genome Sequencing. *J Hum Genet*. **67**, 643–649, doi:10.1038/s10038-022-01066-5. (2022)
55. Yang, X.-L., Bai-Cheng, X., Chen, X.-J., Pan-Pan, B., Jian-Li, M., Xiao-Wen, L. *et al.* Common Molecular Etiology of Patients with Nonsyndromic Hearing Loss in Tibetan, Tu Nationality, and Mongolian Patients in the Northwest of China. *Acta Otolaryngol*. **133**, 930–934, doi:10.3109/00016489.2013.795288. (2013)
56. Li, J.N., Han, D.Y., Hong, M.D. Cochlear implants in post-lingual deafness patients with long-term total hearing loss. *Chinese Journal of Otology*, **8**, 376–381 (2010)
57. Guo, Y.-F., Liu, X.-W., Xu, B.-C., Zhu, Y.-M., Wang, Y.-L., Zhao, F.-F. *et al.* Analysis of a Large-Scale Screening of Mitochondrial DNA m.1555A>G Mutation in 2417 Deaf-Mute Students in Northwest of China. *Genet Test Mol Biomarkers*. **14**, 527–531, doi:10.1089/gtmb.2010.0020. (2010)
58. Liu, X.W., Guo, Y.F., Han, D.Y. [Mitochondrial DNA A1555G mutation analysis in 802 nonsyndromic hearing im-pairment patients]. *Zhonghua Er Bi Yan Hou Tou Jing Wai Ke Za Zhi*, **42**, 739-42. Chinese. (2007)
59. Wang, J., Xiang, J., Chen, L., Luo, H., Xu, X., Li, N. *et al*. Molecular Diagnosis of Non-Syndromic Hearing Loss Patients Using a Stepwise Approach. *Sci Rep*. **11**, 4036, doi:10.1038/s41598-021-83493-6. (2021)
60. Zeng, B., Xu, H., Yu, Y., Li, S., Tian, Y., Li, T. *et al*. Increased Diagnostic Yield in a Cohort of Hearing Loss Families Using a Comprehensive Stepwise Strategy of Molecular Testing. *Front Genet*. **13**, 1057293, doi:10.3389/fgene.2022.1057293. (2022)
61. Liu, X.-W., Wang, J.-C., Wang, S.-Y., Li, S.-J., Zhu, Y.-M., Ding, W.-J. *et al.* The Mutation Frequencies of GJB2, GJB3, SLC26A4 and MT-RNR1 of Patients with Severe to Profound Sensorineural Hearing Loss in Northwest China. *Int J Pediatr Otorhinolaryngol*. **136**, 110143, doi:10.1016/j.ijporl.2020.110143. (2020)
62. Li, Y., Su, J., Zhang, J., Pei, J., Li, D., Zhang, Y. *et al.* Targeted Next-Generation Sequencing of Deaf Patients from Southwestern China. *Molecular genetics & genomic medicine*. **9**, doi:10.1002/mgg3.1660. (2021)
63. Ma, J., Ma, X., Lin, K., Huang, R., Bi, X., Ming, C. *et al*. Genetic Screening of a Chinese Cohort of Children with Hearing Loss Using a Next-Generation Sequencing Panel. *Hum Genomics*. **17**, 1, doi:10.1186/s40246-022-00449-1. (2023)
64. Wu, C.-C., Tsai, C.-Y., Lin, Y.-H., Chen, P.-Y., Lin, P.-H., Cheng, Y.-F. *et al.* Genetic Epidemiology and Clinical Features of Hereditary Hearing Impairment in the Taiwanese Population. *Genes (Basel)*. **10,** 772, doi:10.3390/genes10100772.( 2019)
65. Duan, S., Guo, Y., Chen, X., Li, Y. Genetic mutations in patients with nonsyndromic hearing impairment of minority and Han Chinese ethnicities in Qinghai, China. *J Int Med Res*. **49**, 3000605211000892. doi: 10.1177/03000605211000892. (2021)
66. Usami, S., Abe, S., Akita, J., Namba, A., Shinkawa, H., Ishii, M. *et al*. Prevalence of Mitochondrial Gene Mutations among Hearing Impaired Patients. *J Med Genet*. **37**, 38–40, doi:10.1136/jmg.37.1.38. (2000)
67. Noguchi, Y., Yashima, T., Ito, T., Sumi, T., Tsuzuku, T., Kitamura, K. Audiovestibular findings in patients with mitochondrial A1555G mutation. *The Laryngoscope.* **114**, 344-348. (2004)
68. Mutai, H., Watabe, T., Kosaki, K., Ogawa, K., Matsunaga, T. Mitochondrial Mutations in Maternally Inherited Hearing Loss. *BMC Med Genet*. **18**, 32, doi:10.1186/s12881-017-0389-4. (2017)
69. Usami, S.I. *et al.* The genetic etiology of hearing loss in Japan revealed by the social health insurance-based genetic testing of 10K patients. *Hum Genet*. **141**, 665-681. doi: 10.1007/s00439-021-02371-3. (2022)
70. Bae, J.W., Lee, K.Y., Choi, S.Y., Lee, S.H., Park, H.-J., Kim, U.-K. Molecular Analysis of Mitochondrial Gene Mutations in Korean Patients with Nonsyndromic Hearing Loss. *Int J Mol Med*. **22**, 175–180. (2008)
71. Bae, J.W., Kim, D.-B., Choi, J.Y., Park, H.-J., Lee, J.D., Hur, D.G. *et al.* Molecular and Clinical Characterization of the Variable Phenotype in Korean Families with Hearing Loss Associ-ated with the Mitochondrial A1555G Mutation. *PLoS One.* **7**, e42463, doi:10.1371/journal.pone.0042463. (2012)
72. Malik, S.G., Pieter, N., Sudoyo, H., Kadir, A., Marzuki, S. Prevalence of the Mitochondrial DNA A1555G Mutation in Sensorineural Deafness Patients in Island Southeast Asia. *J Hum Genet*. **48**, 480–483, doi:10.1007/s10038-003-0056-9. (2003)
73. Han, J.J., Nguyen, P.D., Oh, D.-Y., Han, J.H., Kim, A.-R., Kim, M.Y. *et al*. Elucidation of the Unique Mutation Spectrum of Severe Hearing Loss in a Vietnamese Pediatric Population. *Sci Rep.* **9**, 1604, doi:10.1038/s41598-018-38245-4. (2019)
74. Padma, G., Ramchander, P.V., Nandur, V.U., Kumar, K.R., Padma, T. Novel Mutations Affecting the Secondary Structure of MT-RNR1 Gene: A Causal Relationship with Profound Nonsyndromic Hearing Impairment. *Genet Test Mol Biomarkers.* **16**, 1092–1097, doi:10.1089/gtmb.2012.0036. (2012)
75. Subathra, M., Ramesh, A., Selvakumari, M., Karthikeyen, N.P., Srisailapathy, C.R.S. Genetic Epidemiology of Mitochondrial Pathogenic Variants Causing Nonsyndromic Hearing Loss in a Large Cohort of South Indian Hearing Impaired Individuals. *Ann Hum Genet*. **80**, 257–273, doi:10.1111/ahg.12161. (2016)
76. Amritkumar, P., Srisailapathy, C. S. Screening for Mitochondrial A1555G Mutation among Assortative Mating Hearing Impaired Families in South India: Some Vital Insights. *New Frontiers in Medicine and Medical Research*. **1**, 17-27. (2021)
77. Kaheel, H., Breß, A., Hassan, M.A., Shah, A.A., Amin, M., Bakhit, Y.H.Y. *et al.* Frequency of mitochondrial m.1555A > G mutation in Syrian patients with non-syndromic hearing impairment. *BMC Ear Nose Throat Disord*. **21**, 18:7. doi: 10.1186/s12901-018-0055-2. (2018)
78. Khalifa Alkowari, M., Girotto, G., Abdulhadi, K., Dipresa, S., Siam, R., Najjar, N. *et al*. GJB2 and GJB6 Genes and the A1555G Mitochondrial Mutation Are Only Minor Causes of Nonsyndromic Hearing Loss in the Qatari Population. *Int J Audiol*. **51**, 181–185, doi:10.3109/14992027.2011.625983. (2012)
79. Baysal, E., Bayazit, Y.A., Ceylaner, S., Alatas, N., Donmez, B., Ceylaner, G. *et al*. GJB2 and Mitochondrial A1555G Gene Mutations in Nonsyndromic Profound Hearing Loss and Carrier Frequencies in Healthy Individuals. *J Genet.* **87**, 53–57, doi:10.1007/s12041-008-0007-5. (2008)
80. Tekin, M., Duman, T., Boğoçlu, G., İncesulu, A., Çomak, E., Fitoz, S. *et al.* Frequency of MtDNA A1555G and A7445G Mutations among Children with Prelingual Deafness in Turkey. *Eur J Pediatr*. **162**, 154–158, doi:10.1007/s00431-002-1129-z. (2003)
81. Cirçir, Y.E., Incesulu, A., Tekin, M. [Screening of the mitochondrial 12S rRNA (MTRNR1) gene in probands with sensorineural hearing loss]. *Kulak Burun Bogaz Ihtis Derg*. **17**, 75–80. (2007)
82. Dowlati, M.A., Derakhshandeh-Peykar, P., Houshmand, M., Farhadi, M., Shojaei, A., Fallah, M. *et al*. Novel Nucleotide Changes in Mutational Analysis of Mitochondrial 12SrRNA Gene in Patients with Nonsyndromic and Aminoglycoside-Induced Hearing Loss. *Mol Biol Rep*, **40**, 2689–2695, doi:10.1007/s11033-012-2355-8. (2013)
83. Fassad, M.R., Desouky, L.M., Asal, S., Abdalla, E.M. Screening for the Mitochondrial A1555G Mutation among Egyptian Patients with Non-Syndromic, Sensorineural Hearing Loss. *Int J Mol Epidemiol Genet.* **5**, 200–204. (2014)
84. Nahili, H., Charif, M., Boulouiz, R., Bounaceur, S., Benrahma, H., Abidi, O. *et al*. A. Prevalence of the Mitochondrial A1555G Mutation in Moroccan Patients with Non-Syndromic Hearing Loss. *Int J Pediatr Otorhinolaryngol*. **74**, 1071–1074, doi:10.1016/j.ijporl.2010.06.008. (2010)
85. Trotta, L., Iacona, E., Primignani, P., Castorina, P., Radaelli, C., Del Bo, L. *et al.* GJB2 and MT-RNR1 Contributions in Children with Hearing Impairment from Northern Cameroon. *Int J Audiol.* **50**, 133–138, doi:10.3109/14992027.2010.537377. (2011)
86. Adeyemo, A., Faridi, R., Chattaraj, P., Yousaf, R., Tona, R., Okorie, S. *et al*. Genomic Analysis of Childhood Hearing Loss in the Yoruba Population of Nigeria. *Eur J Hum Genet*. **30**, 42–52, doi:10.1038/s41431-021-00984-w. (2022)
87. Kabahuma, R.I., Ouyang, X., Du, L.L., Yan, D., Hutchin, T., Ramsay, M. *et al.* Absence of GJB2 Gene Mutations, the GJB6 Deletion (GJB6-D13S1830) and Four Common Mitochondrial Mutations in Nonsyndromic Genetic Hearing Loss in a South African Population. *Int J Pediatr Otorhinolaryngol*. **75**, 611–617, doi:10.1016/j.ijporl.2011.01.029. (2011)
88. Dahl, H.H.M. *et al.* Etiology and audiological outcomes at 3 years for 364 children in Australia. *PLoS One*. **8**, e59624, doi:10.1371/journal.pone.0059624 (2013)
89. Vandebona, H., Mitchell, P., Manwaring, N., Griffiths, K., Gopinath, B., Wang, J.J. *et al.* Prevalence of mito-chondrial 1555A>G mutation in adults of European descent. *N Engl J Med*. **5**, 642-4. doi: 10.1056/NEJMc0806397. (2009)
