# Supplementary figures and images for: High prevalence of m.1555A > G in patients with hearing loss in the Baikal Lake region of Russia as a result of founder effect
Source: Sci Rep. 2024 Jul 3;14:15342. doi: 10.1038/s41598-024-66254-z (PMC11222474; doi:10.1038/s41598-024-66254-z)

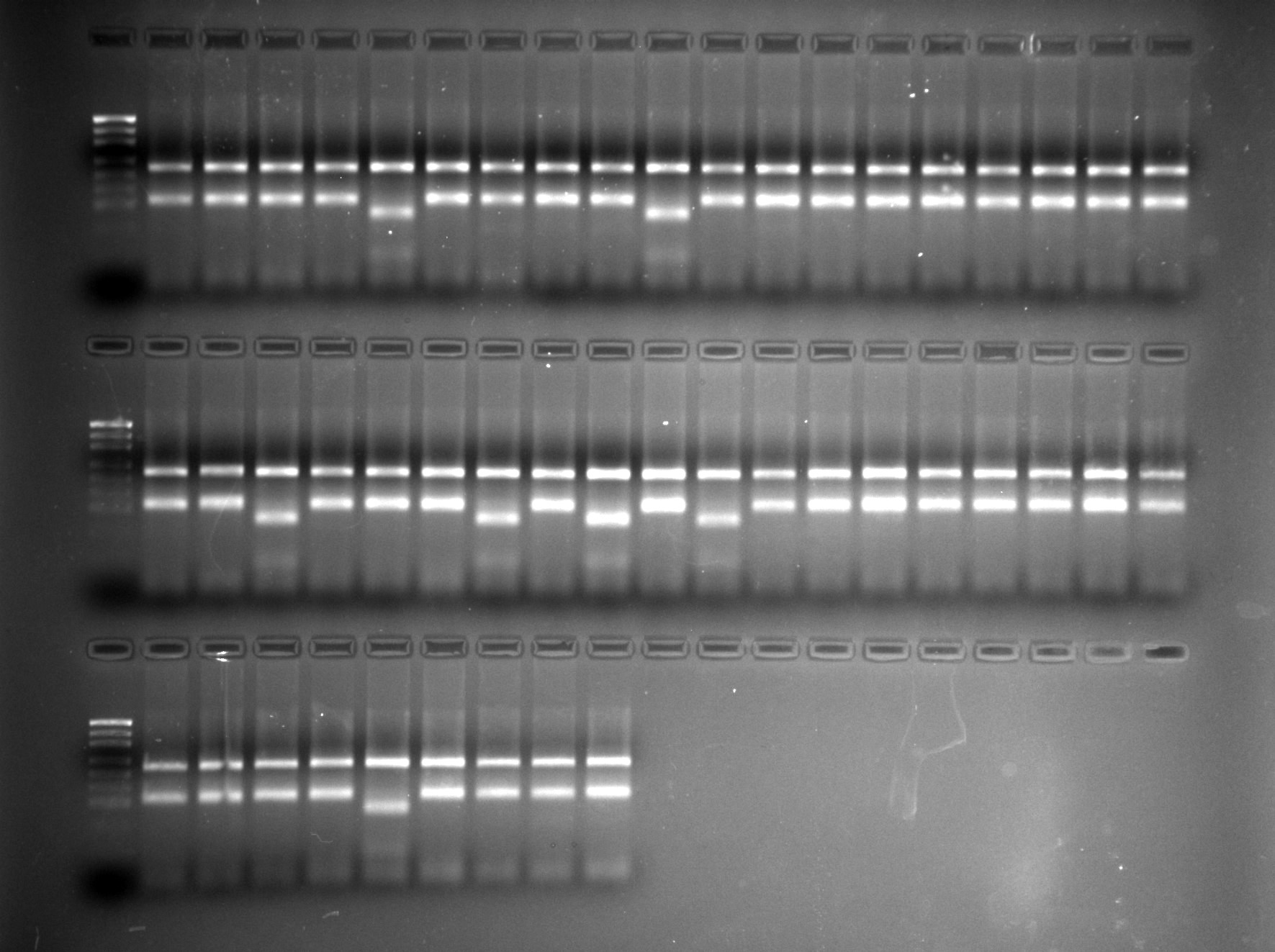

Supplement: Supplementary file 3 — Supplementary Information 3. [file 41598_2024_66254_MOESM3_ESM.tiff]
